# Development of Rheumatoid Arthritis Classification from Electronic Image Sensor Using Ensemble Method

**DOI:** 10.3390/s20010167

**Published:** 2019-12-27

**Authors:** Ho Sharon, Irraivan Elamvazuthi, Cheng-Kai Lu, S. Parasuraman, Elango Natarajan

**Affiliations:** 1 Smart Assistive and Rehabilitative Technology (SMART) Research Group, Department of Electrical and Electronic Engineering, Universiti Teknologi PETRONAS, 32610 Bandar Seri Iskandar, Malaysia; sharon_17005219@utp.edu.my (H.S.); chengkai.lu@utp.edu.my (C.-K.L.); 2 School of Engineering, Monash University Malaysia, 46150 Bandar Sunway, Malaysia; s.parasuraman@monash.edu; 3 Faculty of Engineering, Technology and Built Environment, UCSI University, 56000 Kuala Lumpur, Malaysia; elango@ucsiuniversity.edu.my

**Keywords:** wearable sensor, image sensor, machine learning, medical datasets, ensemble method, classification

## Abstract

Rheumatoid arthritis (RA) is an autoimmune illness that impacts the musculoskeletal system by causing chronic, inflammatory, and systemic effects. The disease often becomes progressive and reduces physical function, causes suffering, fatigue, and articular damage. Over a long period of time, RA causes harm to the bone and cartilage of the joints, weakens the joints’ muscles and tendons, eventually causing joint destruction. Sensors such as accelerometer, wearable sensors, and thermal infrared camera sensor are widely used to gather data for RA. In this paper, the classification of medical disorders based on RA and orthopaedics datasets using Ensemble methods are discussed. The RA dataset was gathered from the analysis of white blood cell classification using features extracted from the image of lymphocytes acquired from a digital microscope with an electronic image sensor. The orthopaedic dataset is a benchmark dataset for this study, as it posed a similar classification problem with several numerical features. Three ensemble algorithms such as bagging, Adaboost, and random subspace were used in the study. These ensemble classifiers use k-NN (K-nearest neighbours) and Random forest (RF) as the base learners of the ensemble classifiers. The data classification is accessed using holdout and 10-fold cross-validation evaluation methods. The assessment was based on set of performance measures such as precision, recall, F-measure, and receiver operating characteristic (ROC) curve. The performance was also measured based on the comparison of the overall classification accuracy rate between different ensembles classifiers and the base learners. Overall, it was found that for Dataset 1, random subspace classifier with k-NN shows the best results in terms of overall accuracy rate of 97.50% and for Dataset 2, bagging-RF shows the highest overall accuracy rate of 94.84% over different ensemble classifiers. The findings indicate that the efficiency of the base classifiers with ensemble classifier have substantially improved.

## 1. Introduction

Arthritis implies irritation of joints. Rheumatoid Arthritis (RA) is a typical type of joint pain. RA is a persistent provocative ailment that affects and decimates the joints of wrists, fingers, and feet. If left untreated, one can lose their ability to lead a normal life. Around 1 out of 100 individuals succumb to RA at a particular phase of their life. It could occur to anybody. RA is not an inherited illness. RA can emerge regardless of age; however, most generally begins around the age of 40 and 60. It is around three times more typical in women than in men. Today, about 1–2% of the world’s population are affected by RA. In a 10-year range of analysis, 50% of patients have extreme impairment and additionally a reduced life span from 3 to 18 years [1]. Over the years, soft computing has increasingly played an important role in helping ailment analysis in doctors’ decision process. The main aim of this study is to investigate the possibility of applying machine learning techniques to the analysis of RA characteristics.

Some of the feasible implementations with the ensemble machine learning include information on-request systems, smart homes control and monitoring systems, mobile games communication interfaces and hospital and outpatient care applications [2,3]. In addition, ensemble machine learning is also used in the development and evaluation of electroencephalogram (EEG)-based brain activity measured by biosensors [4]. Advances in sensor innovation in on-body wearable sensors and pressure sensors have enabled them to be used effectively for the classification of RA disease. Wearable glove sensor based on IOT was used to detect RA disease in [5] was proven to be reliable. Besides, thermal infrared camera sensors were used to sense the temperature changes in between finger joints in a study carried out in [6,7].

Machine Learning (ML) is a research area of computer science that aims to construct predictive data models using algorithms, techniques, and procedures to discover unexpressed associations within the data and create tools that exploit such combinations of description, prediction or prescription [7]. Machine learning was introduced in multiple medical areas and has demonstrated to be very precise in categorizing and detecting illnesses. Machine learning has become a commonly used technique in order to enhance medical service and disease diagnosis with the development of medical information in the medical sector [8]. In order to convert data into information, data mining has become a significant tool. The implementation of data mining in nearly all areas has proven to be effective. The implementation in the medical field has also proven to be comparable. Knowledge obtained through the use of data mining from medical information provides doctors with an extra base of awareness to decide on their procedures, therapy scheduling, risk analytics, and other projections.

Multidiscipline data mining, such as machine learning, statistics, model identification, visualization, etc., includes different methods such as relationship, correlation, optimization, making predictions, classification and clustering [9,10,11,12]. Classification and clustering are the two most commonly used information mining methods. Classification is a type of supervised learning technique with the aim of predicting the target group in the medical data where the classes are predefined for each event. On the other hand, clustering is an unsupervised teaching method, which aims to group information into communities of comparable items in which the items are comparable to each other and not similar items in other groups [13]. Researchers are currently able to use multiple classification algorithms in the literature; hence, it is difficult to choose the finest model for a specific dataset, as these traditional algorithms are subject to issues like computer simplicity, adherence to local minimum standards or overfitting with information for teaching purposes [14].

The ensemble learning is used mainly to enhance a classifier’s efficiency and is one of the most used methods for overcoming these issues. It is a process through which multiple classifiers can be combined in order to categorize new samples to improve prediction accuracy [15]. The most common methods used together are boosting, bagging, stacking, and voting. In the past, studies have demonstrated the use of machine learning algorithms such as k-Nearest Neighbours (k-NN), MultiLayer Perceptron (MLP) and Support Vector Machine (SVM) for classifying and predicting diseases [16,17,18]. Ensemble methods with bagging and adaboost techniques can improve the accuracy of classification substantially by combining various weak classifiers. Ensemble learning on different datasets was successfully evaluated and validated appropriately [17,18].

The study outlined in this article emphasizes the classification of medical data using different ensemble classification techniques such as Bagging, Adaboost and Random subspace together with the base classifiers such as k-NN and RF. In this research, two medical datasets have been used, where the first dataset is rheumatoid arthritis dataset from [19]. The second dataset is from the UCI Machine Learning Repository [8]. This UCI dataset is about Biomechanical features of orthopaedic patients (orthopaedics) presented into two groups with six numerical attributes. Both datasets are classifications of individuals into two classes. Classifications were assessed in terms of accuracy, precision, recall, F-measure and receiver operating characteristic (ROC) curve.

The paper is organized as following: Section 2 discusses the literature review in relation to the work involved. The proposed method and dataset are explained in Section 3. Section 4 presents the results and discussion of the experiment. Finally, Section 5 concludes the paper.

## 2. Literature Review

### 2.1. Related Work

RA is the weakening condition that, if diagnosed at an early stage, could be managed and controlled. This is the only strategy available at present for preventing permanent joint damage or severe deformity. The main issue in RA diagnosis is that RA does not have a particular indicative feature [20], furthermore, the long lag between infection and symptom, and analysis is because of late referral [21]. Das, R and Sengur, A [17] have reported that they were able to diagnose valvular heart disease using ensemble methods, with three classifiers as base classifier, namely, k-Nearest Neighbours (k-NN), Multi-Layer Percentron (MLP) and Support Vector Machine (SVM). The result showed that ensemble classifier can produce better results than single classifier in terms of accuracy. It can be concluded that with base classifier MLP, boosting method has a better performance compared to a single classifier, whereby the sensitivity of 97.3% and specificity of 94% were obtained. In the work by Shiezadeh, et al. [18], algorithm such as ADAboost with J48 learner was chosen as the base learner, decision tree and CSboost were applied to the dataset of 18 attributes. It is proven in his research that CSboost is able to provide the highest accuracy among the classification algorithms. Among the 18 attributes, it was found that gender, number of joints, elbow and knee joint and express sequence tag (EST) test result play the most important roles in the identification of rheumatoid arthritis. Its classification efficiency is as follows: sensitivity of 44% and accuracy of 85%. Early detection of RA and commencement of vigorous treatment during the early stage of the disease can decrease disease development and, in a few patients, even prompt medication free reduction [22]. Ogungbemile [22] classified rheumatoid arthritis (RA) into level of severity using Machine Learning focusing on Decision Tree. This experiment used the temperature dataset extracted from the joints of 18 control subjects and 13 patients that suffered from RA. Then, these datasets were used as the input to the C.5 software to create a classifier. The ratio of Min and Max measurements is the best discriminator, as it is able to generate a sensitivity up to 96% and specificity of 92%. When a person initially has joint torments, it might at first hard for a specialist to state that the individual certainly has RA. This is because there are numerous different reasons for joint torments. There is no single test which determined early RA to have 100% accurateness. Practically speaking, the determination of RA depends on the subject’s history, physical exam, radiographs, and results from the laboratory. This means developing an easy-to-use, non-invasive, and cost-effective diagnostic tool that bridges the gap and makes early diagnosis possible for everyone. The development of this technique has unlimited potential, can also be used as a telemedicine tool, and can therefore, provide diagnostic options for those living in remote areas where the community could not afford advanced equipment such as magnetic resonance imaging (MRI) and high-resolution ultrasound (HRUS). Given the cost-effectiveness of this technology, it can also be used in developing countries, where RA diagnostic tools currently do not exist, given the costly nature of some current images, such as MRI and HRUS.

The diagnosis of RA from a physician is based on symptoms shown by patients. This information can be collected either in the structure of a medical unit or in a patient’s records. This information can comprise of significant details [23], which can be useful. Several techniques and algorithms are being used to collect concealed data. For the past few years, different algorithms of machine learning have been applied on various medical data sets to predict and classify various diseases from medical datasets [24]. Chen et al. [25] used electronic medical record (EMR) data from rheumatology clinic visits to predict RA disease activity. From their study, SVMs with linear kernel were able to deliver the foremost strong performance. It is said to be the best performing model because on average, the 10-fold AUC (Area under Curve) was 0.831 with standard deviation of 0.0317. Furthermore, Carroll et al. [26] have performed analysis of a randomly selected datasets of 400 patients from Vanderbilt University Medical Center’s Synthetic Derivative and Northwestern medical Enterprise Data Warehouse (EDW) using electronic health records, natural language processing (NLP) systems, and codified data. They have classified the data by using Logistic regression algorithm. From their work, it was concluded that, after retraining the model obtained from Partners Healthcare with logistic regression method, the area under the receiver operating curve was found to be 92% for Northwestern and 95% for Vanderbilt compared with 97% obtained at Partners. However, a higher specificity of 97% was obtained compared with 72% obtained at Partners. Das et al. [27] reported that using distinctive attribute correlated to the pathology can clearly define classification of fibromyalgia and rheumatoid arthritis. The results have shown that using ADAboost classifier is able to achieve a success rate of 89%, while reaching 97.8596% in the best case. Research by Ku Abd. Rahim et al. [2] showed that SVM with ensemble classifiers produced the best overall accuracy rate of 99.22% for classification of human daily activities using ensemble methods. Research reported by Nurhanim [3] provided the best overall classification rate of 98.57% with Multiclass support vector machine polynomial kernel (MC-SVM-Polynomial) for classification of daily physical activities such as walking, sitting, standing, laying, walking upstairs and downstairs. In general, the conclusion is that the ensemble methods produce better results in comparison with other algorithms.

Supervised learning checks for the relations between a number of examples (input functions) and one or more defined targets (output variables) and then produce a model that predicts the output correctly when there is new set of unlabelled input data. The training data should be marked with the right input-output combinations for supervised learning to work. Supervised learning comprises both classifications, which involve predicting the class or category provided with a new set of input (therefore the result is a discreet variable), and regression, where a continuous variable value is to be estimated for a new sample. Classification and regression are types of supervised learning. As an example, in order for researchers to determine whether the treatment response of patients treated with special therapy can be predicted by a series of clinical features, a supervised learning algorithm may be applied to datasets in which each patient record contains a set of clinical features of interest, as well as a label that shows the degree of disease response. Another word for supervised learning is to learn from examples. This paper focuses on the classification method from the supervised learning. Classification techniques such as Naïve Bayesian classification, support vector machines (SVM), decision trees, logistic and linear regression, random forests and k-nearest neighbours are based on supervised learning algorithms [28,29,30].

### 2.2. Sensing for RA

Diagnosis and sensing of RA is a challenging proposition because the associated clinical features are broad, and onset is generally subtle. The American College of Rheumatology (ACR) [31] has prescribed criteria for the characterization of RA (the purported “ACR 1987 revised criteria”). The disease usually affects the metacarpophalangeal, wrist, and proximal interphalangeal joints. Non-invasive imaging technologies such as MRI, radiography, ultrasound, and thermal imaging are used for the diagnosis of RA. Researchers from [19] tried to investigate RA by automating the procedure with the use of lymphocytes in blood cell pictures. Patients may suffer without apparent joint swelling at early stages of the disease, at which radiography fails to show [22]. In addition, magnetic resonance imaging (MRI) can distinguish aggravation and joint swelling; however, it is costly and not realistic for redundant utilization [32]. Another technique, ultrasound, showed more prominent dependability and affectability in the locating of synovitis and emanation as to clinical examination [32]. The computed tomography (CR) is an important tool for the detection of structural bone abnormalities and the reduction of the articulated area [33]. However, this method could not detect the earliest stages of synovitis of the soft tissue and therefore reducing the rheumatologist’s information on the activity of the disease [34]. Lastly, infrared, also known as thermal imaging, is a technique that uses thermographic camera to capture invisible infrared images, and those images are converted to normal images that are visible to the human eye. Many different thermal imaging systems have been tested over the years since dedicated medical thermographs have been available.

#### 2.2.1. Definition of RA

**Figure 1:**
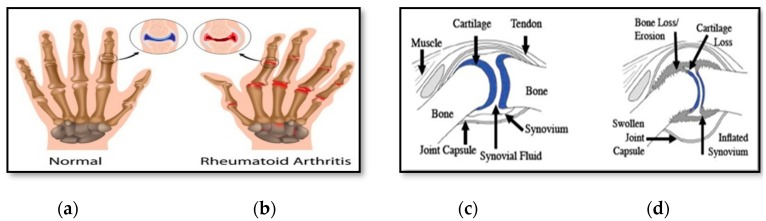
(**a**) Shows an example of normal joints in the hand, highlighting the location of a proximal interphalangeal joints (PIP) joint, (**b**) shows the inflamed joints of the hands with RA, highlighting the location of a PIP joint, (**c**) is an illustration of a typical healthy joint, and (**d**) an illustration of a typical joint exhibiting symptoms of RA. Figure 1 (**c**,**d**) also shows the schematic of a typical synovial joint.

Rheumatoid Arthritis (RA) is a persistent provocative ailment that effects and decimates the joints of wrists, fingers, and feet. If left untreated, one can lose their ability to lead a normal life [1]. A joint is a structure at which two parts of the bones are fitted together. Joints permit development and adaptability of different parts of the body. Muscles that take out on ligaments that are appended to bone caused the motion of the bones. The edge of bones is covered by the ligaments. Synovial liquid, which is a thick liquid, is located between the ligaments of two bones that make up a joint. The liquid smoothens up the joint, which permits continuous operation among the bones. The synovium is known as the tissue that encompasses a joint. The cell of the synovium produces the synovial liquid. Capsule is known as external piece of the synovium. It is hard, provides the joint steadiness, and prevents the bones from moving out of joint [35]. Encompassing tendons and muscles additionally help to provide support and solidness to joints. RA is categorized as an autoimmune illness. Synovitis is a key characteristic of RA and is directly linked to multiple alterations to the underlying joint physiology. The differences between a healthy synovium and an inflamed synovium are strikingly clear from histological analysis. Figure 1 shows the effects of synovium on healthy and typical joint exhibiting symptoms of RA.

The revised criteria for classification of RA was based on the definitions of the ACR 1987 [31]. Based on this definition, a subject is said to have RA on the off chance that he or she has fulfilled no less than four of the seven criteria for no less than a month and a half.

#### 2.2.2. Sensors for Detecting RA

**Figure 2:**
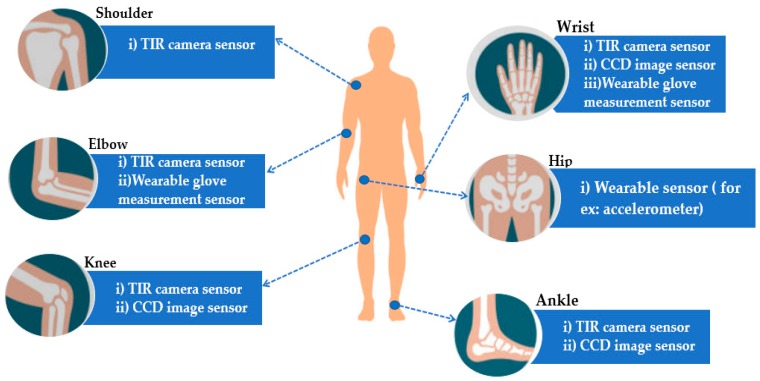
Sensor used for each joint.

There are different sensor elements being used to capture the data. The various types of sensors are used to acquire data to diagnose RA disease are shown in Figure 2.

The authors of previous research [5,6] have used thermal infrared camera (TIR) imaging system as a sensor to diagnose RA. This non-invasive thermal imaging instrument has demonstrated the ability in the assessment and diagnosis of RA. TIR imaging may render a diagnosis without physical symptoms feasible because it is a temperature measurement over a certain joint showing as an RA indicator. A characteristic of RA is inflammation in the articulations affected, which can easily be seen by thermal imaging, but not by the naked eye, as the temperature rises automatically around those articulations. The two authors [5,6] used TIR sensor for different parts of the body. From [5], the author used TIR sensor to acquire image analysis of the elbows, wrist and hand joints, knees, ankles and feet joints. Furthermore, authors in [7] used TIR sensor to capture the image of finger joints.

Moreover, the author of [36] used the acquisition of optical pictures using a CCD (charge-coupled device) image sensor from the side of the dorsal hand. In order to detect optical modifications, a non-tomographic, single-wavelength, CW transillumer has earlier been provided, in which a light source focused on the finger’s side, using a CCD camera sensor to collect the images on the palmar side.

The author from [37] used a wearable sensor on the wrist in terms of accelerometer to access the pain and stiffness experienced by the patients. The research shows that regular, sensor based, controlled, unmonitored physical exams can be feasible and useful to objectively evaluate the effect of the illness on home environment function. Lightweight tiny accelerometers, generally worn on the wrist, hip or upper arm, allow for a constant recording over several days of information relating to movements (e.g., trunk, wrist, or ankle accelerations). The patients are required to perform an unsupervised version of a standard motor task, known as the Five Times Sit to Stand (5 × STS) test. This study has proved that the usage of sensor based, and unsupervised physical tests definitely has an impact in diagnosing this disease in a home environment.

Researchers in [5] captured the real time data from wearable glove measurement sensor and a 3D interface. This system will accurately quantify patients accurately in grades, maximum and minimum joint range, flexure, extension, and abduction of the finger or thumb joints, and compares the joint range of the normal ROM values in order to detect the deformity of the hand and rigidity of moveable finger joint.

## 3. Methods

### 3.1. Proposed Model

**Figure 3:**
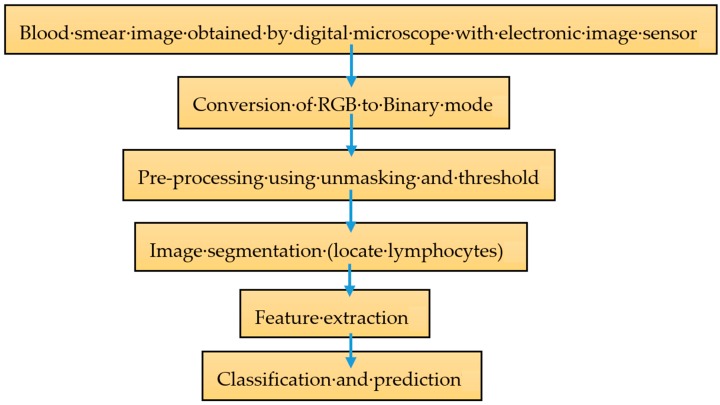
Steps in Methodology.

The methodology carried out in this research is defined in the following steps in Figure 3. RA can reduce the level of lymphocytes. Lymphocytopenia, a medical term used for a low count of white blood cells may be a RA complication called Felty syndrome that presents signs of severe pain, morning stiffness and joint swelling. Automatic assessment of lymphocytes requires a strong algorithm of segmentation that identifies the blood smear picture lymphocytes. Precision is essential as the classification depends on these segmentation steps. Analysis of the classification of white blood cells using features extracted from lymphocyte images is very effective.

According to Figure 3, blood smear images collected under 100× microscope with an effective 1000 magnification. As for RA, only lymphocytes must be taken as the region of interest (ROI) and the vital characteristics must be taken from it. Generally, digital microscopes generate pictures in (red, green and blue) RGB color space. In order to make the segmentation easier, RGB images are then converted to binary. Pre-processing is an important step in removing noise from the images. Next, the goal is to segment the images from white blood cell (WBC) and lastly to separate lymphocytes from the background. Feature extraction is the steps to discover features from the image. Then, classification would be carried out using Ensemble classifier. Ensemble classification method includes two main processes, which are training and testing. The Ensemble classifier’s goal, based on the training dataset, is to deliver a decision model that can predict the test information class label given by the feature vector. Moreover, algorithm could be tuned accordingly to produce a better accuracy of the prediction model. This experiment is using the base classifier method consist of k-NN and Random Forest to generate classifier models together with ensemble classifiers of bagging, boosting and random subspace. The assessment criteria for this classification algorithm includes accuracy, precision, recall, F-measure, and AUC that can be found from the confusion matrix output.

### 3.2. Database

Two datasets were used in this study. Dataset 1 is obtained from [19]. Dataset 1 was collected using lymphocyte in the blood cell images acquired from microscopic image. This dataset contains 40 instances, each containing 9 attributes named area, perimeter, circularity, integrated density, Median, Skewness, raw integrated density, and Roundness and solidity. Therefore, in total, 360 data (40 × 9) were used.

Dataset 2 is from the UCI Machine Learning Repository [8]. This UCI dataset comprises of Biomechanical features of orthopaedic patients (orthopaedics) consists 6 attributes and 310 instances named pelvic tilt numeric, lumbar lordosis angle, pelvic incidence, pelvic radius, sacral lope, lumbar lordosis angle, and degree spondylolisthesis. Dataset 2 is a benchmark dataset for this study, as it posed a similar classification problem with several numerical features which were provided for the classification. Models were run first on the orthopaedics dataset to ensure they were correctly implemented. Every entry in the Datasets 1 and 2 corresponds to a different patient.

### 3.3. Data Pre-Processing

This is a significant preliminary phase in the data mining method involving cleaning, assimilation, reconstruction, extraction and selection of features [38]. The precision and reliability of the assessment is determined by the data quality. The information may contain missing values, noise, irrelevance and redundancy. The data mining process will result in misleading results if data is not handled properly. The RA used in this study has 5 missing values. Missing data can be handled in many different ways. For this approach, the RA dataset was refined by replacing the records containing missing value with the mean value of the variable. Data pre-processing therefore functions as a preliminary method in which information can be transformed for clustering.

### 3.4. Classification Techniques—Proposed Methods

Recent developments in computer learning have led to methods that improve the performance of basic learning systems or enhance the capabilities of them. These learning systems have been called as ‘ensembles’. Ensembles are machine-learning processes that uses the ability from several models to obtain greater prediction than any model can on its own [39]. The algorithm building the model is called an inducer and an inducer is regarded as a classifier for certain training sets [40]. An ensemble consists of several inducers, frequently known as the base classifier. A base learner is an algorithm which gets a number of labelled cases as an input and generates a model to simplify those cases. Predictions for new unclassified instances are determined using the model created. The ensemble classification shows the use of multiple classifier combinations. These combinations operate in three steps. The first step is to construct multiple training subsets from a training set. In second step, each classifier is built exclusively on the basis of both the algorithm and the subset of data training. In the final step, base classifier outcomes are incorporated, and ultimate outcomes are acquired in a greater tier phase called ensemble classifier. It is known that an ensemble often performs far better than the individual classifiers which form it. In this study, two medical datasets were evaluated with ensemble methods such as Bagging, Adaboost, and Random Subspace. An ensemble inducer can be of any sort of base classifiers such as k-NN, decision tree, linear regression and other type of base learner algorithm. In this research work, the base learners used were k-NN and RF. An overview of the ensemble methods can be discovered in [41] for bagging, [42] for Adaboost and [43] for Random subspace.

The three ensemble methods and three basic learner algorithms described above were used to classify the medical datasets based on a data mining tool known as the WEKA 3.9 version [44], using a holdout model assessment system (containing 70% of training set and 30% of test set) and a 10-fold cross-validation method.

### 3.5. Performance Measures

The performance measures used in this study are based on the following terms:

(1) $$\mathrm{Accuracy}=\frac{TP+TN}{TP+TN+FP+FN}$$

(2) $$\mathrm{Precision}=\frac{TP}{TP+FP}$$

(3) $$\mathrm{Recall}=\frac{TP}{TP+FN}$$

(4) $$F-\mathrm{measure}=\frac{2\left(TP\right)}{2\left(\left(TP\right)+\left(FN\right)\right)}$$

where *TP*—True Positive, *TN*—True Negative, *FP*—False Positive, and *FN*—False Negative.

The AUC is a Receiver Operating Curve (ROC) curve scalar value which is used to explore different model’s performance. The AUC measures the model’s ability to distinguish between distinct class values. It is a graphical strategy for showing the trade-off between a classifier’s true positive rate (recall) and false positive rate. The true positive rate is also referred to as recall in Equation (3) and false positive rate is defined as the following term:

(5) $$\mathrm{False}\;\mathrm{positive}\;\mathrm{rate}=\frac{FP}{FP+TN}$$

## 4. Results and Discussion

### 4.1. Performance Measures of Dataset 1

#### 4.1.1. 10-Fold Cross-Validations for Precision, Recall, F-Measure and ROC Measures

**Figure 4:**
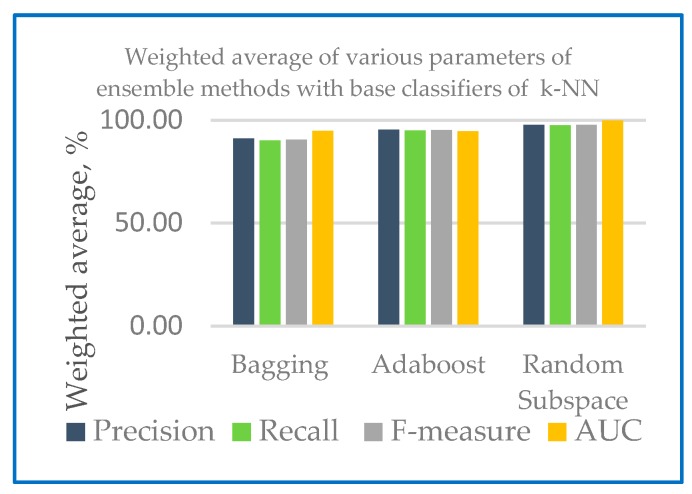
Weighted average of various parameters of ensemble methods with base classifiers of k-NN.

**Figure 5:**
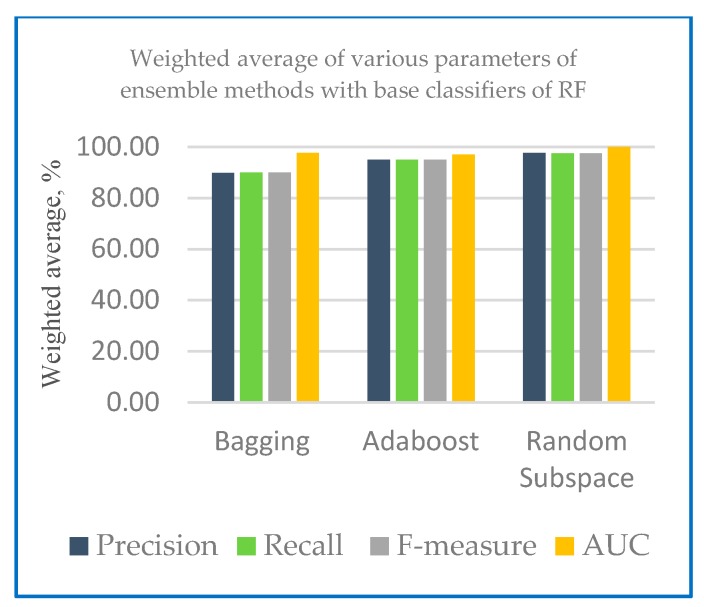
Weighted average of various parameters of ensemble methods with base classifiers of random forest.

The performance measures of different ensemble machine learning algorithms together with two base classifiers k-NN and RF are shown in the following section. Table 1 shows the performance measures of the developed ensemble model with k-NN and random forest as base classifier. Figure 4 shows the weighted average of various parameters of ensemble methods with base classifiers of k-NN and Figure 5 shows the weighted average of various parameters of ensemble methods with base classifiers of random forest.

Figure 4 and Figure 5 show that in the case of all test configurations, the metric values were very similar. The results of random subspace classifier with k-NN and random forest as base classifier on the 10-fold cross-validation method perform equally well with precision and recall with 97.60% and 97.50 respectively compared to another ensemble classifier. Higher recall percentage can diagnose the correct disease for the patient. With base classifier of k-NN and RF, Adaboost and random subspace gives a higher precision result range of 97.60% to 95.30% compared to Bagging with 89.80% to 91.10%. In the case of recall, the percentage lies in the range of 97.50% to 95.00% for Adaboost and random subspace compared to bagging with 90.00%. Excellent results were gained in terms of F-measure for both base classifiers (most of the values are over 90%). From Table 2, all of the models developed AUC statistics are above 0.90. This means that all models perform more than 90%. The AUC results for k-NN were from 94.70% to 99.70% and 97.00% to 100% for RF. Table 2 shows an example of variability score of Dataset 1 using combination of random subspace with k-NN as base classifier.

Table 3 shows the AUC value for Dataset 1 with k-NN as the base classifier with the three-ensemble classifier, bagging, boosting and random subspace using cross validation method.

**Figure 6:**
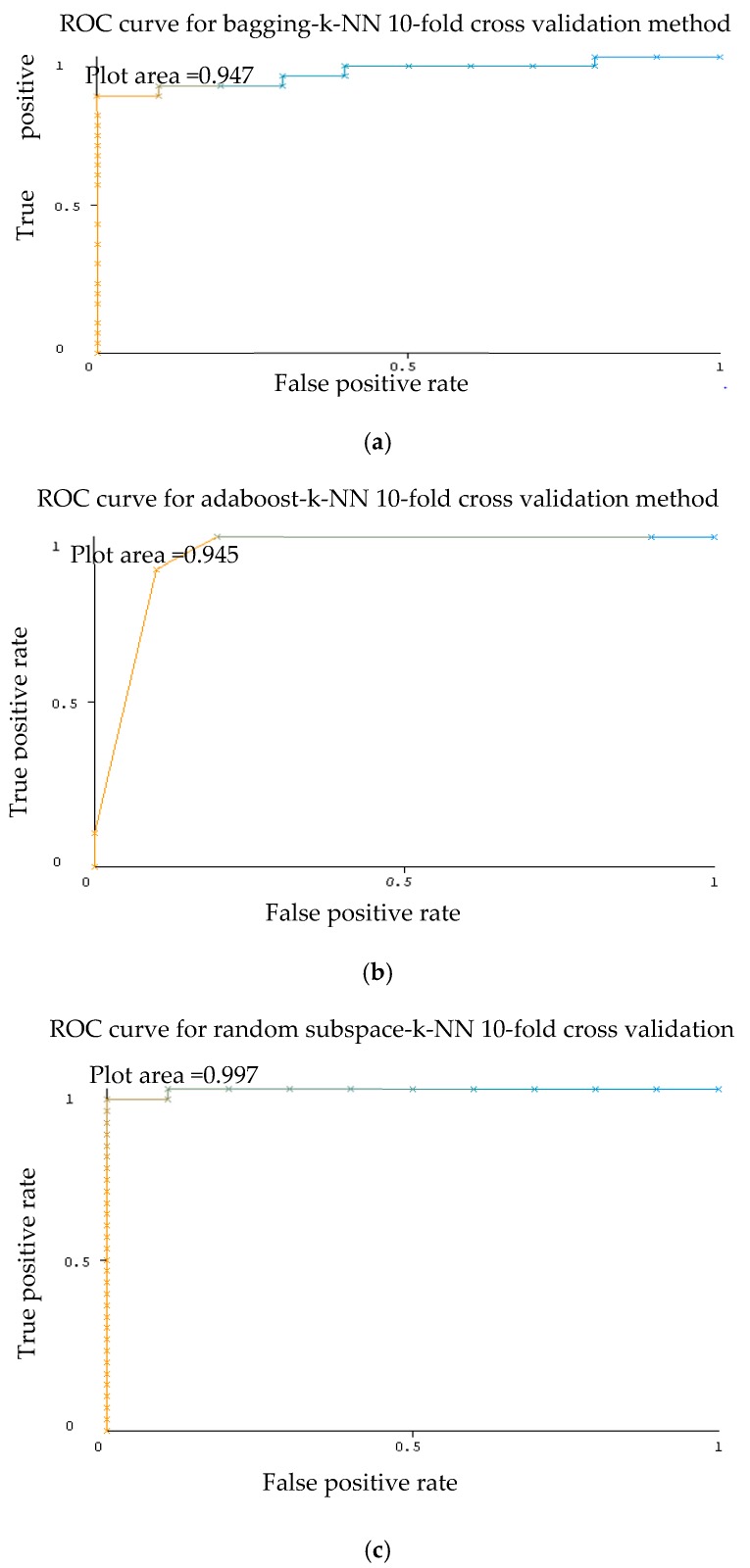
Receiver operating characteristic (ROC) graph activity of ensemble classifier bagging (**a**), Adaboost (**b**), random subspace (**c**) with k-NN classifier using 10-fold cross validation method Dataset 1.

As shown in Table 3, excellent results were gained for the AUC for each ensemble classifier with k-NN as the base classifier. As stated in the literature, the model is perfect when its area under the curve is equal to 1, whereas the model makes a random assessment when its area below the curve is equivalent to 0.5. In this case of 3 ensemble classification with k-NN as base classifier, AUC value seems to be close to 1 (Plot area = 0.947) which indicates an accurate classification. Representative ROC curves for k-NN are displayed in Figure 6 based on Table 3.

#### 4.1.2. Holdout Method for Precision, Recall, F-Measure and ROC Measures

**Figure 7:**
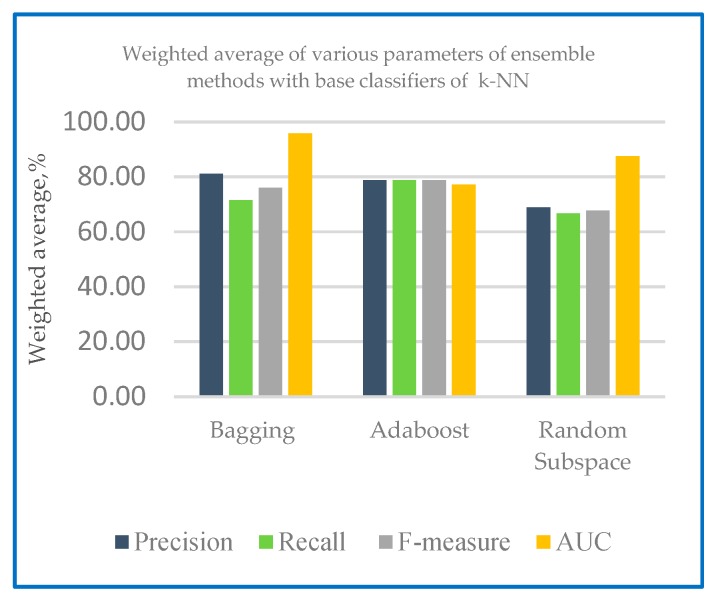
Weighted average of various parameters of ensemble methods with base classifiers of k-NN.

**Figure 8:**
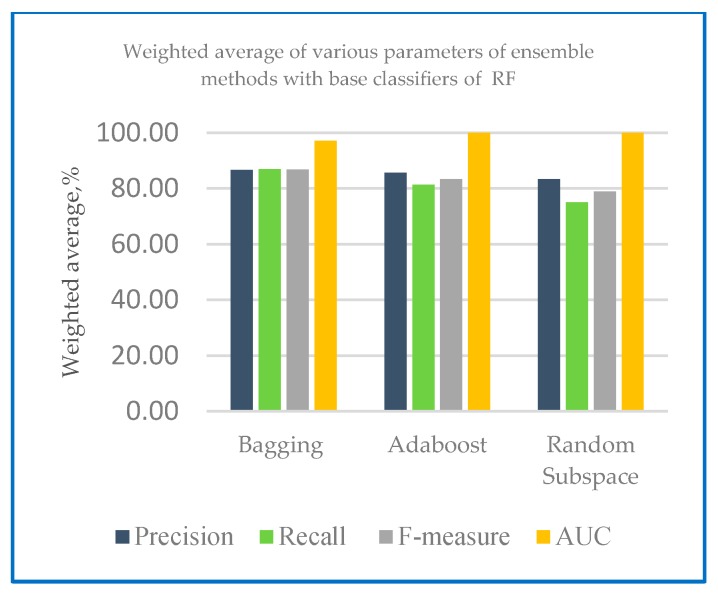
Weighted average of various parameters of ensemble methods with base classifiers of RF.

Table 4 shows the performance measures with base classifiers of k-NN and RF on holdout method, Figure 7 shows the weighted average of various parameters of ensemble methods with base classifiers of k-NN and Figure 8 shows the weighted average of various parameters of ensemble methods with base classifiers of RF.

From Table 4, the results of ensemble classifier using holdout method indicates RF as the base learner generates a better precision of results ranging from 83.30% to 86.60% compared to k-NN with 68.80% to 81.00%. Results of recall evaluation shows that RF as base learner gained greater range of results from 75.00% to 95.80%. The results show that the RF base classifier with ensemble algorithm has the highest value for both precision and recall. Thus, it has the highest value for F-measure ranging from 78.93% to 86.80%. RF with ensemble algorithm is found to have an enhanced value of ROC with results ranging from 97.10% to 100% compared to k-NN with 77.10% to 95.80%. Table 5 shows the ROC curve for Dataset 1with k-NN as the base classifier with the three-ensemble classifier, bagging, boosting and random subspace using holdout method.

**Figure 9:**
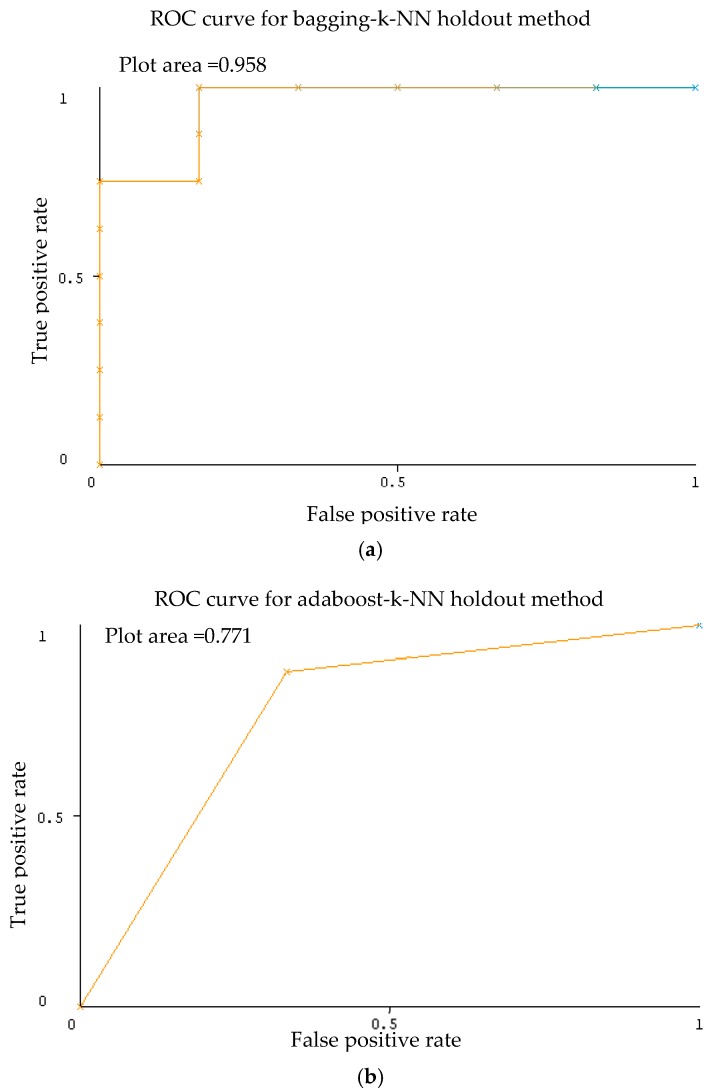

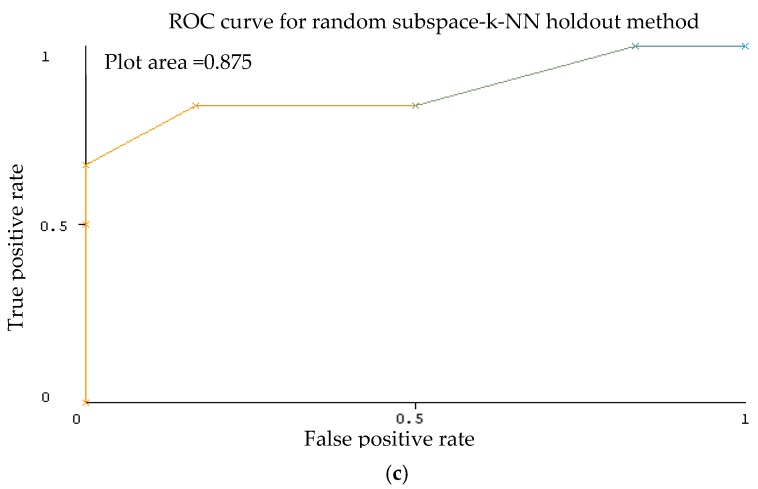
ROC graph activity of ensemble classifier bagging (**a**), Adaboost (**b**), random subspace (**c**) with k-NN classifier using holdout method Dataset 1.

As shown in Table 5, the results for the AUC evaluation with k-NN as the base classifier for bagging recorded the best AUC value of 0.958. Representative ROC curves for k-NN are displayed in Figure 9 based on Table 5.

#### 4.1.3. 10-Fold Cross Validation and Holdout for Overall Accuracy Rate

**Figure 10:**
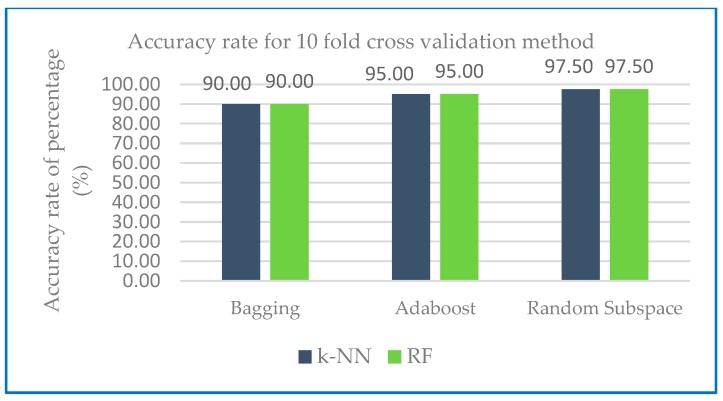
Accuracy rate of Dataset 1 with 10- fold cross validation method.

**Figure 11:**
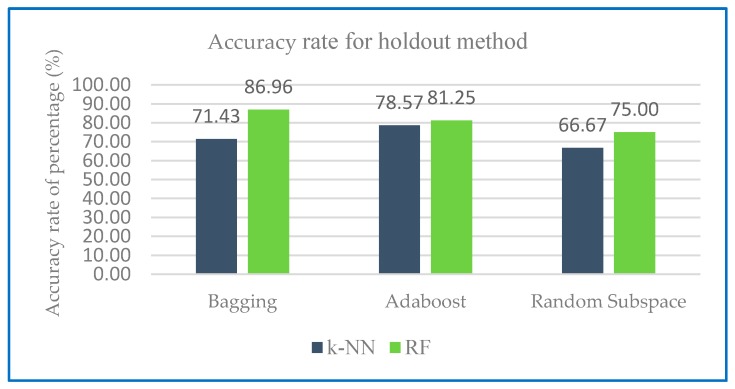
Accuracy rate of Dataset 1 with holdout method.

Figure 10 and Figure 11 shows the accuracy rate classification for Dataset 1 for ensemble method with base classifier k-NN and RF using 10-fold cross validation and holdout method.

As shown in Figure 10, the accuracy rate of ensemble classifier with the two base classifier k-NN and RF have the same level of performance. Using bagging ensemble classifier, accuracy rate: 90.00%, with Adaboost ensemble, accuracy rate: 95.00% and random subspace ensemble, accuracy rate: 97.50%. Figure 10 predict that random subspace performs in an excellent manner with k-NN and RF as the base classifier, while all other ensemble classifiers performs well. All test configurations delivered models that classified more than 90% of instances correctly.

The accuracy rate of ensemble methods with base learners k-NN and RF 10-fold cross validation method for Dataset 1 have also been presented in the Table 6 for better visualization.

Results in Table 6 presented that all of the testing configurations gained very good results. In the case of base classifier, k-NN and RF perform equally well with the same accuracy rate for all ensemble classifier. It is important to note that in the case of the ensemble classifier random subspace with both base classifier k-NN and RF, the accuracy is significantly improved compared to ensemble classifier bagging. The accuracy difference between ensemble classifier bagging compared to random subspace is equals to 7.5. Figure 11 shows the Accuracy rate of Dataset 1 with holdout method.

Results from Figure 11 predict the accuracy rate for respective ensemble classifier with RF using holdout method is higher with a range of 75.00% to 86.96% compared to k-NN with range of 66.67% to 71.43%.

The results from the Figure 11 have been also presented in Table 7.

From Table 7, while the classifications in all the ensembles have good performance with base classifier generally, it turns out that base classifier k-NN was a poor performer and RF was the optimal classifier with the ensemble classifier. Overall results of 10-fold cross-validation model evaluation provide better results for both base learners as compared to holdout.

### 4.2. Performance Measures of Dataset 2

#### 4.2.1. 10-Fold Cross Validation Method for Precision, Recall, and ROC Measures

**Figure 12:**
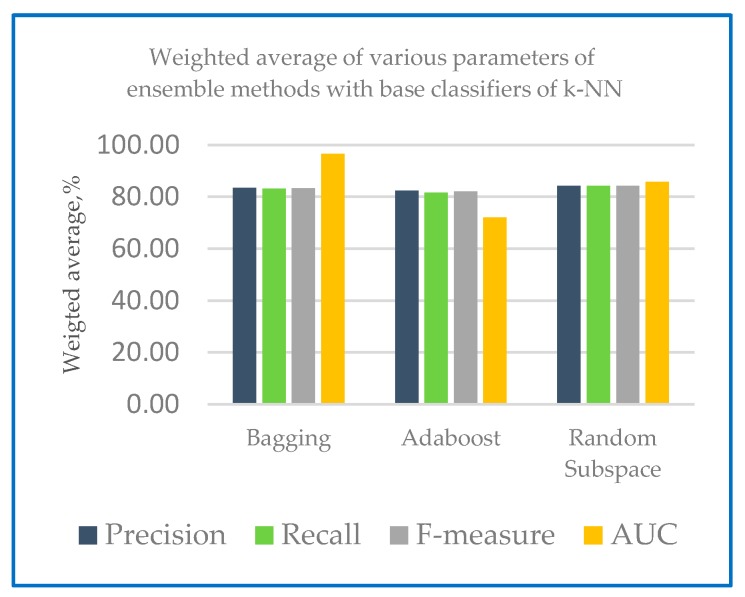
Weighted average of various parameters of ensemble methods with base classifiers of k-NN.

**Figure 13:**
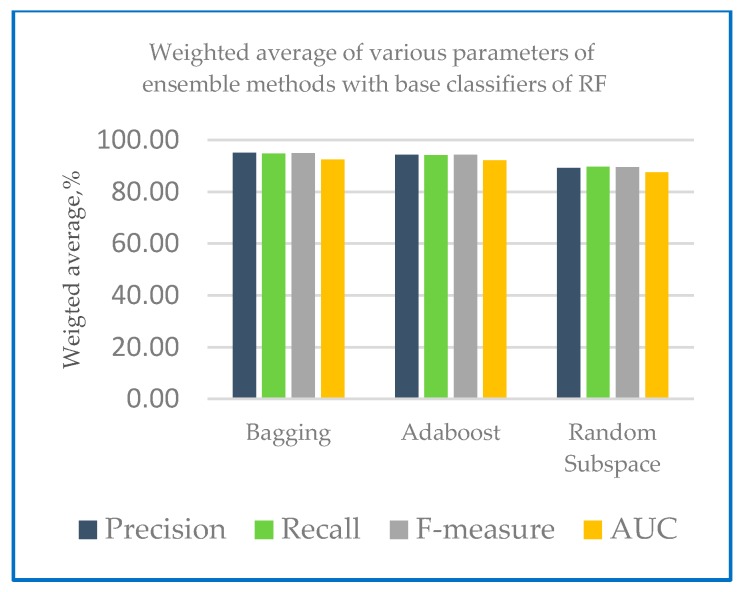
Weighted average of various parameters of ensemble methods with base classifiers of RF.

Table 8 presents the performance measures of the developed ensemble model with k-NN and random forest as base classifier with 10-fold cross validation method. Figure 12 shows the weighted average of various parameters of ensemble methods with base classifiers of k-NN and Figure 13 shows the weighted average of various parameters of ensemble methods with base classifiers of random forest.

From Table 8, the best precision results bagging classifier on 10-fold cross validation method shows that RF gained 95.00% compared to k-NN which gained 83.50%. For other ensemble classifier, Adaboost-k-NN and Adaboost-RF obtained precision of 82.40% and 94.30% respectively compared to random subspace–k-NN and random subspace-RF which recorded 84.20% and 89.20% respectively. Recall results achieved 94.80% using bagging-RF compared to bagging-k-NN which achieved 83.20%. The recall results obtained 84.20% for both random subspace-k-NN and Adaboost-RF. With Adaboost-k-NN, recall recorded 81.60% and random subspace-RF recorded recall result of 89.70%. With k-NN as base classifier, F-measure performance ranging from 82.00% to 84.20%, while with RF as base classifier, F-measure performance ranging from 89.55% to 94.90%. The AUC had high values for all of the testing configurations (AUC_max_ = 92.50%) with RF base classifier. Lower results were gained for the ROC with k-NN base classifier (AUC_min_ = 80.70%)

Table 9 shows the AUC value for Dataset 2 with RF as the base classifier with the three-ensemble classifier, bagging, boosting and random subspace using cross validation method.

**Figure 14:**
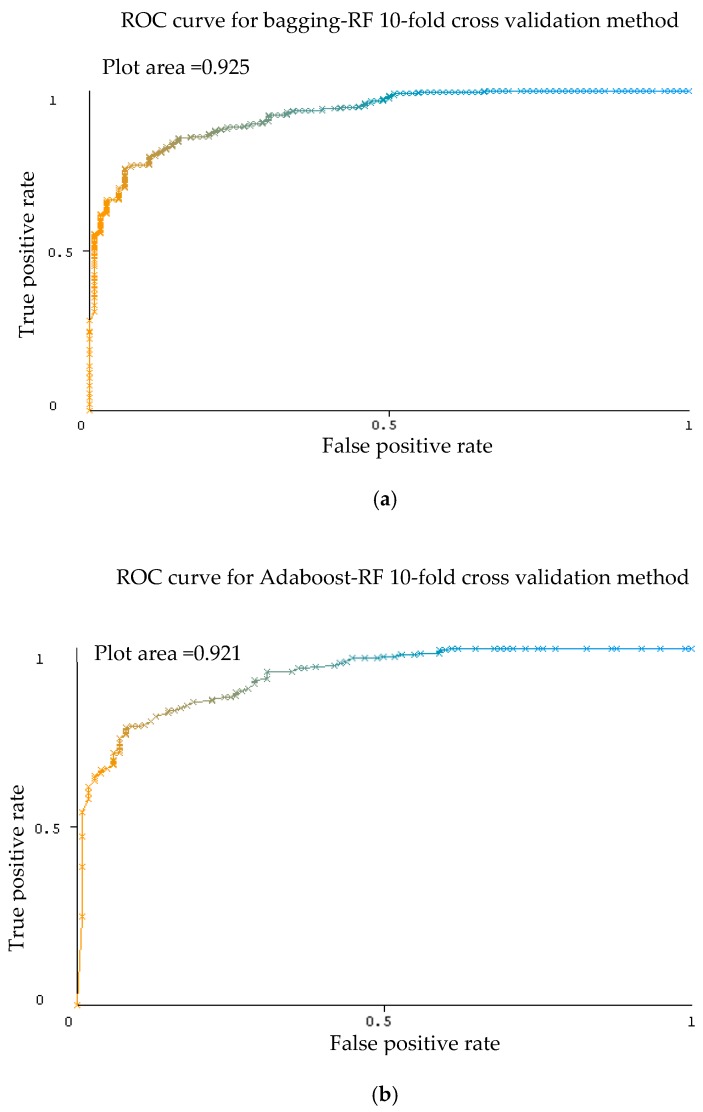

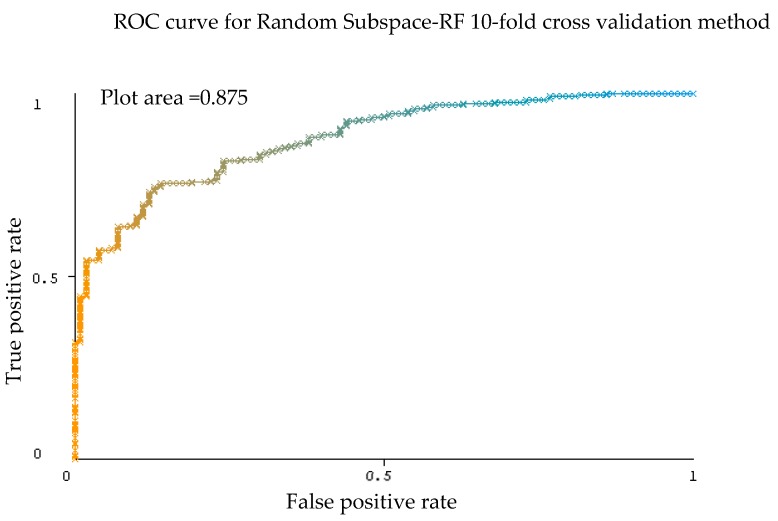
ROC graph activity of ensemble classifier bagging (**a**), Adaboost (**b**), random subspace (**c**) with RF classifier using 10-fold cross validation method Dataset 2.

As shown in Table 9, excellent results were gained for the AUC evaluation for each ensemble classifier with RF as the base classifier. Representative ROC curves for RF are displayed in Figure 14 based on Table 9.

#### 4.2.2. Holdout Method for Precision, Recall, and ROC Measures

**Figure 15:**
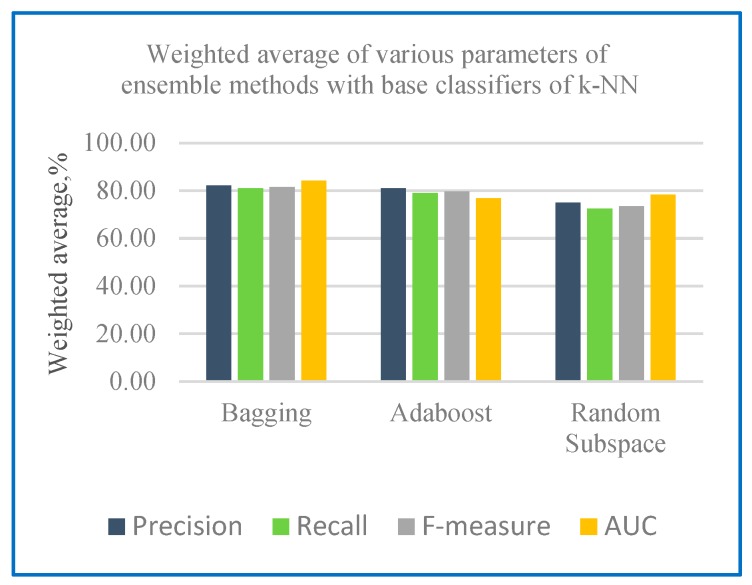
Weighted average of various parameters of ensemble methods with base classifiers of k-NN.

**Figure 16:**
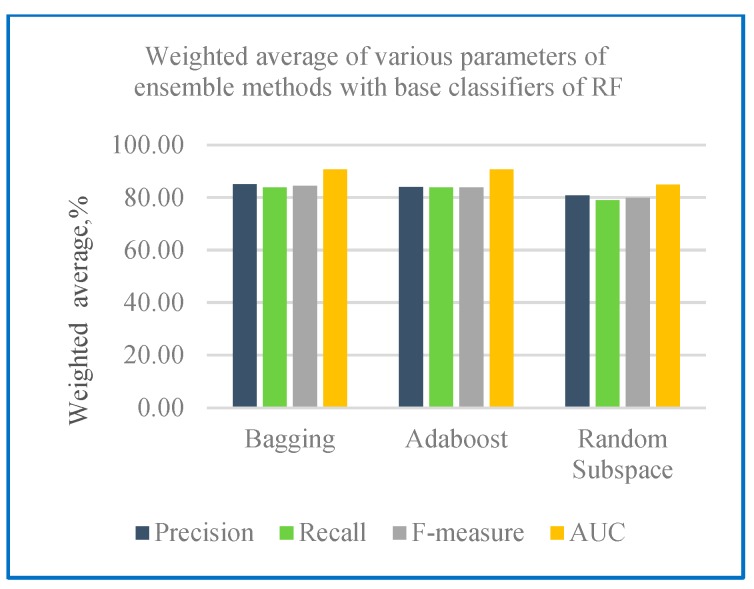
Weighted average of various parameters of ensemble methods with base classifiers of RF.

Table 10 demonstrate the performance measures of developed ensemble model with k-NN and random forest as base classifier with holdout method. The performance measures of ensemble methods with base learners k-NN and RF for Dataset 2 have also been presented in the Figure 15 and Figure 16 for greater visualization.

As demonstrated in Table 10, the results of bagging classifier with k-NN and RF base classifier evaluation on holdout method obtained the best precision with 85.10%(RF) and 82.10%(k-NN) respectively. For other ensemble classifier, Adaboost-k-NN and Adaboost-RF shows a higher precision performance of respectively 80.90% and 84.00% compared to random subspace–k-NN which recorded 75.00% and random subspace-RF 80.90%. For recall, bagging and Adaboost performed similarly as they both obtained same precision value of 83.80%. Random subspace-k-NN obtained a recall of 72.40% and random subspace-RF obtained 79.00% which performance is lower than bagging and Adaboost classifier. The above bar chart reveals that F-measure of the RF base classifier with ensemble classifier ranging is higher than k-NN base classifier. The AUC had high values for all of the testing configurations (AUC_max_ = 90.70%) with RF base classifier. Lower results were gained for the ROC with k-NN base classifier (AUC_max_ = 76.70%).

Table 11 shows the AUC value for Dataset 2 with RF as the base classifier with the three-ensemble classifier, bagging, boosting and random subspace using holdout method. 

**Figure 17:**
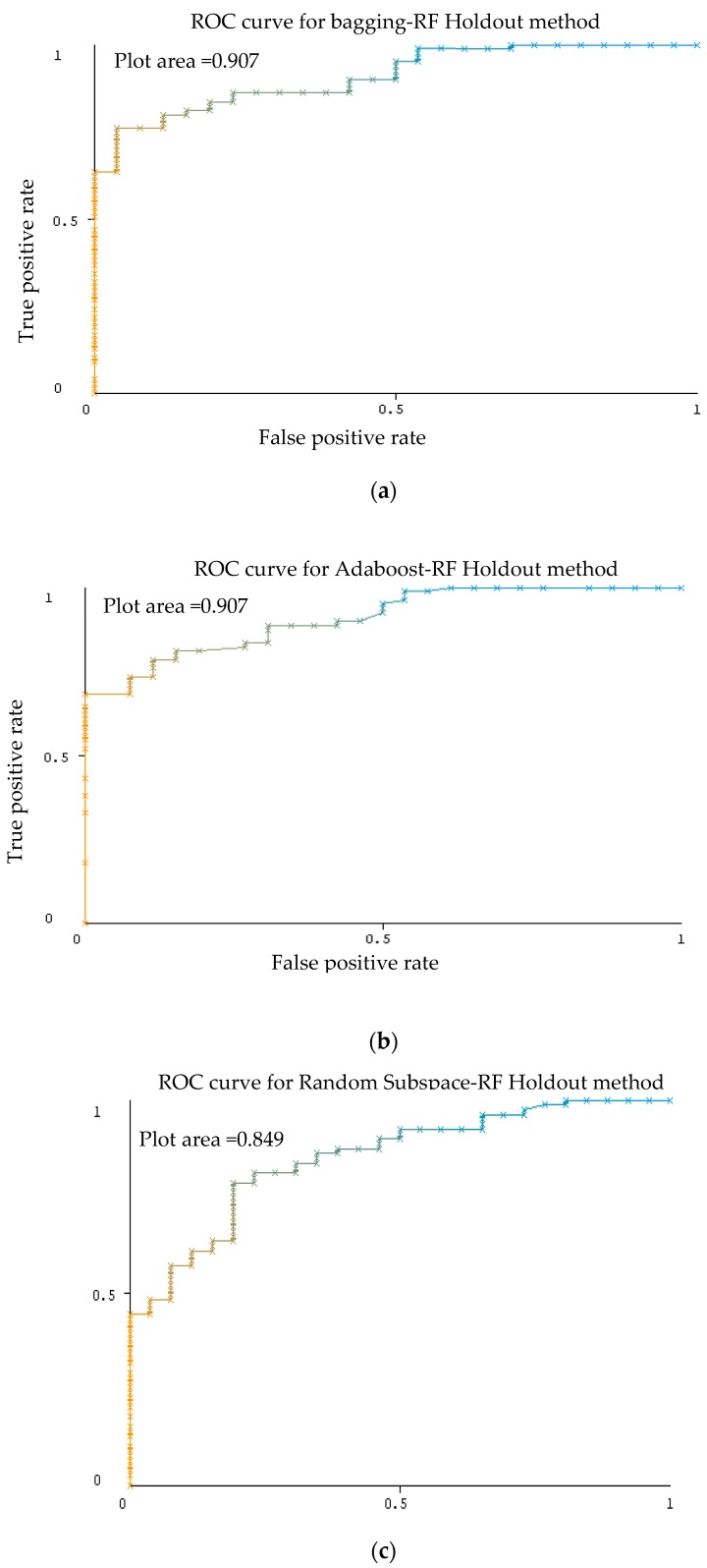
ROC graph activity of ensemble classifier bagging (**a**), Adaboost (**b**), random subspace (**c**) with RF classifier using holdout method Dataset 2.

As shown in Table 11, for each bagging and Adaboost ensemble classifier with RF as the base classifier produced the same results for the AUC evaluation, 0.907. For ensemble random subspace, AUC evaluation recorded 0.849. Representative ROC curves for k-NN are displayed in Figure 17 based on Table 11.

#### 4.2.3. 10-Fold Cross Validation and Holdout for Overall Accuracy Rate

**Figure 18:**
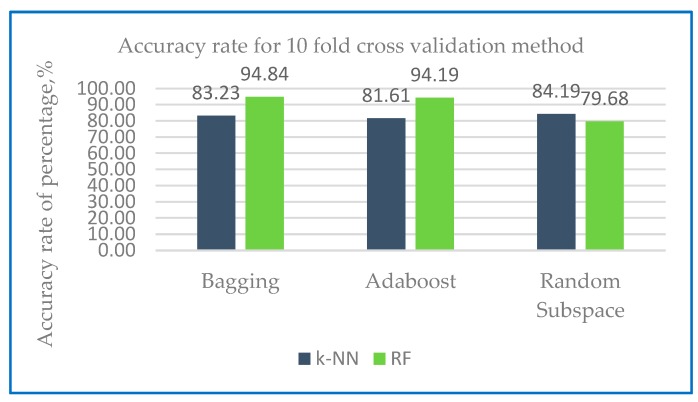
Accuracy rate of Dataset 2 with 10-fold cross validation method.

**Figure 19:**
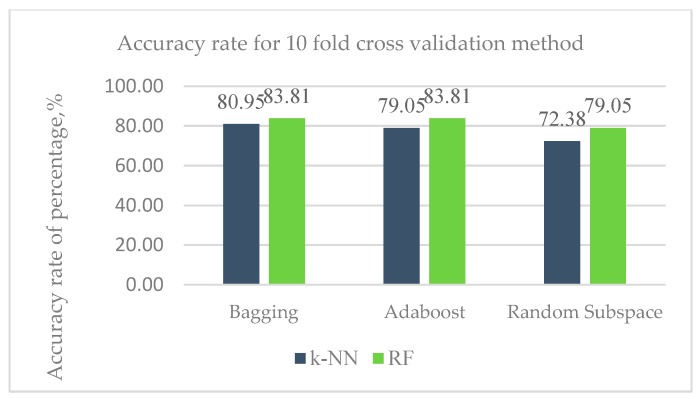
Accuracy rate of Dataset 2 with holdout method.

Figure 18 and Figure 19 show the accuracy rate classification for Dataset 2 for ensemble method with base classifier k-NN and RF using 10-fold cross validation and holdout method.

Figure 18 predict that bagging-RF happens to be the best performer with accuracy of 94.84%. With the base classifier k-NN, the accuracy for ensemble classifier range from 81.61% to 84.19%. On the other hand, Random subspace-RF is found to be a poor performer with the lowest accuracy of 79.68%. With base classifier RF, the accuracy for ensemble classifier range from 79.68% to 94.84%.

The results from Figure 18 obtained with the use of Dataset 2 with 10-fold cross validation method are also shown in Table 12.

Table 12 predict that RF works better with the ensemble bagging and Adaboost with accuracy of more than 84%. On the other ensemble random subspace, RF accuracy performance fluctuated to 79.68%. While with base classifier k-NN, all ensemble classifier accuracy performance is above 80%

Figure 19 shows the Accuracy rate of Dataset 2 with holdout method.

Results from Figure 19 predict the accuracy rate for respective ensemble classifier with RF using holdout method is higher with a range of 79.05% to 83.81% compared to k-NN with range of 72.38% to 80.95%.

The results from the Figure 19 have been also displayed in the Table 13. The figure clearly shows the diversity of values of particular metrics for different ensemble classifier.

Table 13 gives the measurement of accuracy improvement enhancement in regard to the different classifiers considered here. RF with bagging and Adaboost ensemble classifier happens to be the best performer with accuracy of 83.81%. Overall results of 10-fold cross-validation model evaluation provide better results for both base learners as compared to holdout.

### 4.3. Comparative Analysis

The overall classification accuracy rate comparison of the various classification methods with previous work is represented in Table 14.

From Table 14 it is seen that the proposed model for Dataset 1 with random subspace-k-NN obtained an accuracy rate of 97.50 and for Dataset 2 with bagging-RF achieved an accuracy rate of 94.84 using 10-fold cross validation. The proposed model gives an enhanced accuracy result on the same data set that have been used when compared with the previous methods by Chokkalingam and Komathy [19] that has achieved an accuracy rate of 96.50% and Hasan and Islam [8] that obtained an accuracy rate of 92.00%. The proposed model classification accuracy is higher when compared to work done by previous researchers for both Datasets 1 and 2. The proposed model for Dataset 1, random subspace-k-NN has the ability to produce a higher accuracy rate. This should be because of k-NN’s stabilization with regard to training sets alterations. Moreover, k-NN is a highly stable classifier, whereas other ensemble methods like bagging and boosting are developed by sub-sampling the training examples don’t operate well with stable classifiers. This corresponds to the results reported by Skurichina and Duin [45] and Zhi-Hua Zhou [46]. K-NN is benefitted by random subspace, as Sun et al. [47] pointed out, because random subspace is classified in much smaller subspaces, which can decrease adverse noise impact for neighbour computation.

The proposed model bagging-RF for Dataset 2 showed good accuracy rate. Random forest is an improvement of the bagging which improves the range of variable. The random forest classifier combines the benefits of bagging with random selection of functions [48]. This paper is able to validate one of the advantages listed in their paper [49] namely, random forest method has excellent tolerance to noise and great accuracy of classification and the random forest method can be competent in view of the enormous amount of large data.

The result deduced that the proposed model random subspace-k-NN for Dataset 1 and bagging-RF for Dataset 2, the 10-fold cross validation method outperforms the holdout method. Several researchers such as Witten and Frank [24], Bengio et al. [50], Sakr et al. [51], Kim [52] and Dietterich [53] supported this claim that very often, such a cross-validation produces excellent outcomes and is better than any spilt. The k-fold cross validation can be repeated in t-times to reduce the randomness influence caused by the spread of data [46]. It must be noted that the model with high accuracy, recall, precision, ROC and F-measure is the best predictive model and highly desired. Ahmad et al. [54] and Zhi-Hua Zhou [46].

It can be concluded that an ensemble method is often considerably more precise than a single learner, and ensemble techniques in many real-world assignments have already been very successful. There are three threads pointed out by Zhi-Hua Zhou [46] that have led to the present area of ensemble methods: the combination of classifiers, ensemble of weak learners and mixture of experts Ensemble of weak learners was mostly researched in the community of machine learning. In this field, the researches focused on the weak learners and create powerful algorithms to enhance the performance from weak to strong [55]. This work by the researches has resulted to the development of well-known ensemble techniques such as Adaboost and Bagging. In general, most ensemble methods for the type of base classification used to construct the ensemble are self-determined. This is an important benefit that enable a specific classifier to be developed which could be best known for a particular application.

## 5. Conclusions

In this research, the usage of different ensemble classifiers with different base learner algorithms was implemented for different medical data sets. This study was conducted on rheumatoid arthritis and biomechanical features of orthopaedics datasets. The efficiency and comparison of the algorithms is also done using three popular ensemble classifiers: bagging, boosting, and random subspace with base learner such as k-NN and RF. The experimental results also showed that ensemble classifier is dependent on the type of base classifiers and type of data used. Moreover, this study also deduced, there are selected classifiers that should be used for each dataset. Several performance measures were utilized to evaluate the effectiveness of the classification techniques such as overall accuracy, recall, precision, ROC and F-measures. There are two evaluation methods, holdout and 10-fold cross validation methods were used to identified to yield the best model. The results of the comparison algorithms show that for Dataset 1, random subspace classifier with k-NN shows the best results in terms of overall accuracy rate compared to different ensemble classifiers. On the other hand, for Dataset 2, bagging-RF shows the highest overall accuracy rate over different ensemble classifiers. The comparison in the classification of both datasets shows the overall results of performance for recall, precision, ROC and F-measures shows a slight tendency favouring 10-fold cross validation method. It is concluded that using ensemble classifiers for diagnosing medical data enhance the classification accuracy, although different ensemble classifier would show different performance. In the future, different approaches and their application to our ensemble approach would be explored, which is crucial for the efficient diagnosis which would enhance the health index.

## Figures and Tables

**Table 1:** Performance measures with base classifiers of k-NN and RF on 10-fold cross-validation method.

	k-NN				Random Forest			
	Precision	Recall	F-Measure	AUC	Precision	Recall	F-Measure	AUC
Bagging	91.10	90.00	90.54	94.70	89.80	90.00	89.90	97.70
Adaboost	95.30	95.00	95.15	94.50	95.00	95.00	95.00	97.00
Random Subspace	97.60	97.50	97.55	99.70	97.60	97.50	97.55	100.00

**Table 2:** Variability score of Dataset 1 of random subspace with k-NN as base classifier.

Fold	Prediction
1	0.862
2	0.864
3	0.781
4	0.723
5	0.888
6	0.783
7	0.888
8	0.783
9	0.848
10	0.874

**Table 3:** Area under Curve (AUC) evaluation of ensemble with k-NN base classifier.

k-NN (10-Fold Cross Validation)	
Ensemble Classifier	AUC
Bagging	94.70
Adaboost	94.50
Random Subspace	99.70

**Table 4:** Performance measures with base classifiers of k-NN and RF on holdout method.

	k-NN				Random Forest			
	Precision	Recall	F-Measure	AUC	Precision	Recall	F-Measure	AUC
Bagging	81.00	71.40	75.90	95.80	86.60	87.00	86.80	97.10
Adaboost	78.70	78.69	78.70	77.10	85.60	81.30	83.39	100.00
Random Subspace	68.80	66.70	67.73	87.50	83.30	75.00	78.93	100.00

**Table 5:** ROC evaluation of ensemble with k-NN base classifier.

k-NN (Holdout)	
Ensemble Classifier	AUC
Bagging	0.958
Adaboost	0.771
Random Subspace	0.875

**Table 6:** Overall performance measure ensemble classifiers for 10-fold cross validation method.

Accuracy Rate		
10-Fold Cross-Validation		
Ensemble Method	k-NN	RF
Bagging	90.00	90.00
Adaboost	95.00	95.00
Random Subspace	97.50	97.50

**Table 7:** Overall performance measure ensemble classifiers for holdout method.

Accuracy Rate		
Holdout		
Ensemble Method	k-NN	RF
Bagging	71.43	86.96
Adaboost	78.57	81.25
Random Subspace	66.67	75.00

**Table 8:** Performance measures with base classifiers of k-NN and RF on 10-fold cross validation method.

	k-NN				Random Forest			
	Precision	Recall	F-Measure	AUC	Precision	Recall	F-Measure	AUC
**Bagging**	83.50	83.20	83.35	89.80	95.00	94.80	94.90	92.50
**Adaboost**	82.40	81.60	82.00	80.70	94.30	94.20	94.25	92.10
**Random Subspace**	84.20	84.20	84.20	87.40	89.20	89.70	89.55	87.50

**Table 9:** AUC evaluation of ensemble with RF base classifier.

RF (10-Fold Cross Validation)	
Ensemble Classifier	AUC
Bagging	0.925
Adaboost	0.921
Random Subspace	0.875

**Table 10:** Performance measures with base classifiers of k-NN and RF on holdout method.

	k-NN				Random Forest			
	Precision	Recall	F-Measure	AUC	Precision	Recall	F-Measure	AUC
Bagging	82.10	81.00	81.54	84.20	85.10	83.80	84.45	90.70
Adaboost	80.90	79.00	79.94	76.70	84.00	83.80	83.90	90.70
Random Subspace	75.00	72.40	73.68	78.20	80.90	79.00	79.94	84.90

**Table 11:** AUC evaluation of ensemble with RF base classifier.

RF (Holdout Method)	
Ensemble Classifier	AUC
Bagging	0.907
Adaboost	0.907
Random Subspace	0.849

**Table 12:** Overall performance measure ensemble classifiers for 10-fold cross validation method.

Accuracy Rate		
10-Fold Cross-Validation		
Ensemble Method	k-NN	RF
Bagging	83.23	94.84
Adaboost	81.61	94.19
Random Subspace	84.19	79.68

**Table 13:** Overall performance measure ensemble classifiers for holdout method.

Accuracy Rate		
Holdout		
Ensemble Method	k-NN	RF
Bagging	80.95	83.81
Adaboost	79.05	83.81
Random Subspace	72.38	79.05

**Table 14:** Overall classification accuracy comparison with previous research work.

Reference	Evaluation Method	Dataset	Classification Method	Overall Accuracy Rate
Proposed model	10-fold cross-validation	Dataset 1 (40 samples)	Random subspace-k-NN	97.50%
Proposed model	Holdout	Dataset 1 (40 samples)	Random subspace-k-NN	66.67%
Proposed model	10-fold cross-validation	Dataset 2 (310 samples)	Bagging-RF	94.84%
Proposed model	Holdout	Dataset 2 (310 samples)	Bagging-RF	83.81
S. P. Chokkalingam and K. Komathy [19]	Cross validation	Dataset 1 (40 samples)	ADT Tree	96.50%
Hasan.K and Islam.S [8]	Holdout	Dataset 2 (310 samples)	Decision Tree	92.00%
